# Non-invasive assessment of coronary endothelial function in children and adolescents with type 1 diabetes mellitus using isometric handgrip exercise—MRI: A feasibility study

**DOI:** 10.1371/journal.pone.0228569

**Published:** 2020-02-13

**Authors:** Gaëtan Zwingli, Jérôme Yerly, Yvan Mivelaz, Sophie Stoppa-Vaucher, Andrew A. Dwyer, Nelly Pitteloud, Matthias Stuber, Michael Hauschild

**Affiliations:** 1 Lausanne University (UNIL), Faculty of Biology and Medicine, Lausanne, Switzerland; 2 Department of Radiology, Lausanne University Hospital (CHUV), Center for Biomedical Imaging, Lausanne, Switzerland; 3 Pediatric Cardiology Unit, Service of Pediatrics, Lausanne University Hospital (CHUV), Lausanne, Switzerland; 4 Department of Pediatrics, Hôpital Neuchâtelois, Neuchâtel, Switzerland; 5 Pediatric Endocrinology, Diabetology and Obesity Unit, Service of Pediatrics, Lausanne University Hospital (CHUV), Lausanne, Switzerland; 6 Boston College, William F.Connell School of Nursing, Chestnut Hill, MA, United States of America; 7 Service of Endocrinology, Diabetology and Metabolism, Lausanne University Hospital (CHUV), Lausanne, Switzerland; University of New South Wales, AUSTRALIA

## Abstract

**Background:**

Type 1 diabetes mellitus (T1DM) in children and adolescents is associated with significant cardiovascular morbidity and mortality. Early detection of vascular dysfunction is key to patient management yet current assessment techniques are invasive and not suitable for pediatric patient populations. A novel approach using isometric handgrip exercise during magnetic resonance imaging (IHE-MRI) has recently been developed to evaluate coronary endothelial function non-invasively in adults. This project aimed to assess endothelium-dependent coronary arterial response to IHE-MRI in children with T1DM and in age matched healthy controls.

**Materials and methods:**

Healthy volunteers and children with T1DM (>5 years) were recruited. IHE-MRI cross-sectional coronary artery area measurements were recorded at rest and under stress. Carotid intima media thickness (CIMT) and aortic pulse wave velocity (PWV) were assessed for comparison. Student’s t-tests were used to compare results between groups.

**Results and discussion:**

Seven children with T1DM (3 female, median 14.8 years, mean 14.8 ± 1.9 years) and 16 healthy controls (7 female, median 14.8 years, mean 14.2 ± 2.4 years) participated. A significant increase in stress-induced cross-sectional coronary area was measured in controls (5.4 mm^2^ at rest to 6.39 mm^2^ under stress, 18.8 ± 10.7%, *p* = 0.0004). In contrast, mean area change in patients with T1DM was not significant (7.17 mm^2^ at rest to 7.59 mm^2^ under stress, 10.5% ± 28.1%, *p* = n.s.). There was no significant difference in the results for neither PWV nor CIMT between patients and controls, (5.3±1.5 m/s vs.4.8±0.7 m/s and 0.4±0.03mm vs.0.46 mm ± 0.03 respectively, both *p* = n.s.).

**Conclusions:**

Our pilot study demonstrates the feasibility of using a totally non-invasive IHE-MRI technique in children and adolescents with and without T1DM. Preliminary results suggest a blunted endothelium-dependent coronary vasomotor function in children with T1DM (>5 years). Better knowledge and new methodologies may improve surveillance and care for T1DM patients to reduce cardiovascular morbidity and mortality.

## Introduction

Type 1 diabetes mellitus (T1DM) is a chronic disease characterized by immune-mediated beta-cell destruction requiring lifelong insulin therapy. T1DM is most often diagnosed in young children (2–3 years-old) and pre-teens/early adolescents (8–12 years-old). The incidence and prevalence of T1DM in children and adolescents <16 years is rising and incidence rates vary across countries [1]. Despite newly emerging treatment modalities [2], high rates of mortality from acute and chronic complications persist [3]. Both disease duration and elevated glycated hemoglobin (HbA1c) levels are associated with increased cardiovascular morbidity and all-cause mortality [4]. The Diabetes Control and Complications Trial (DCCT) has used magnetic resonance imaging (MRI) [5] to investigate cardiovascular effects of DM. The vascular endothelium serves as an important modulator of vasomotor tone and function via the release of nitric oxide. The release of endothelium derived nitric oxide is thought to be necessary to regulate vascular response to blood flow demands during exercise. Early studies showed that exercise training improved endothelium-dependent vasodilatation as measured by changes in coronary artery diameter through serial angiograms in response do intracoronary infusion of increasing doses of acetylcholine [6]. Notably, end-stage lesions (e.g. atherosclerotic plaques) have been observed in children and young adults [7]. Clinical detection of early stages of vascular disease (e.g. endothelial dysfunction and arterial stiffness due to intima media lesion) is therefore paramount, especially in individuals with risk factors such as obesity, hypertension, and a positive familial history. Importantly, the availability and effects of early secondary prevention accentuate the need for non-invasive studies to detect vascular disease in children and young adults.

Both carotid intima media thickness (CIMT) and aortic pulse wave velocity (PWV) are the standard non-invasive methods for investigating vascular effects of diabetes in children and adolescents. Other techniques to directly assess coronary endothelial function often require invasive coronary angiography and cannot be used in healthy subjects and children [8]. Measurement of the carotid intima media thickness (CIMT) with B-mode ultrasonography [9] is one of the most common used methods of vascular imaging studies and has been extensively studied in adults with diabetes [10] and several reports suggest the presence of increased CIMT values in a large proportion of children with T1DM ([11]. Aortic pulse wave velocity (PWV) has also been used to assess aortic wall stiffness [12–14]. Recent studies point to the utility of assessing arterial stiffness by PWV, in children with varied congenital heart and vascular malformations [15–17]. Studies reveal stiffer, less compliant thoracic aortas and impaired arterial stiffness in adolescents with T1DM.

Recently, Hays et al. explored the use of MRI to measure cross-sectional coronary area and blood flow changes in response to isometric handgrip exercise (IHE) in adults [18]. The IHE-MRI technique was previously shown to be useful for assessing the effect of ischemia on myocardial metabolism [19]. Indeed, *in vivo* studies demonstrate the vasomotor response to IHE is primarily mediated by nitric oxide [20] and therefore, reflects endothelial function [6, 21–25]. The feasibility of the IHE-MRI technique to assess vasomotor reactivity in coronary arteries has been studied in patients with coronary artery disease as well as patients with HIV [18, 21, 26]. Phantom study results suggest that radial MRI is capable of distinguishing area differences in the order of 0.2–0.3 mm^2^ (i.e. 3–4% difference for a 3 mm baseline diameter) [27]. Thus, radial MRI is adequate for measuring area differences in the range of previously reported endothelium dependent vasomotor response in healthy adult subjects (10–25%). Further, these results also indicated that the smallest detectable area difference with radial MRI was largely independent of pixel size in the resolution range investigated [27]. Coronary artery diameter ranges from 2 mm in healthy infants to 5 mm in healthy teenagers [28]. However, evaluation of the endothelial function by MRI has, to the best of our knowledge, not been studied in children.

This study aimed to test the feasibility of using IHE-MRI in children and adolescents by assessing endothelial function in young patients with and without T1DM. Secondarily we sought to compare IHE-MRI results with the standard CIMT and PWV techniques.

## Materials and methods

This investigator-initiated, single-center pilot study was an unblinded parallel trial of IHE-MRI versus CIMT and PWV examining blood vessel function in young children (adolescent and pre-adolescent) at the University Hospital of Lausanne (CHUV) in Switzerland.

Prior to study enrollment, patients and their parents/legal representatives received oral and written information about the study. Written informed consent was obtained from all parents/guardians and also from participants over the age of 14 years.

### Study participants

Two groups of children were recruited: patients with T1DM of at least 5 years duration, and healthy community-dwelling children without any ongoing medication.

Participants ranged from 12–18 years of age, exhibiting Tanner II or greater pubertal development. Exclusion criteria included smoking, obesity (BMI >97th percentile), systolic or diastolic hypertension (>90th percentile), dyslipidemia (pathological HDL/total cholesterol), or any known inflammatory process. Participants’ characteristics, such as age, duration of diabetes, insulin therapies and recent Hb1AC levels were recorded for children with T1DM. On the day of the exams, height, weight, heart rate and blood pressure were measured.

### Imaging protocol

Patients with T1DM removed technical material (e.g. insulin pump, continuous glucose measurement (CGM) devices) prior to imaging. This was planned in advance with the families in order to minimize the study impact on T1DM management. Both CIMT and PWV were performed by the same operator (YM) and IHE-MRI by another operator assisted by a technician. Technicians varied according to the hospital schedule. All IHE-MRI analysis were performed by the same investigator (JY). Exams took place in the afternoon, at least one-hour postprandial.

To assess vascular function using CIMT and PVW, subjects were examined by ultrasound using Philips iE33 (Philips Medical, Netherlands) echocardiograph with a linear L11-5 transducer. Data were saved digitally (Xcelera, Philips Medical, Netherlands) and analyzed off-line using an automated measurement program (QLAB software, Philips Medical, Netherlands). Images were acquired following the standards of the American Heart Association (AHA) [29]. IMT of the posterior wall was measured in three portions of the left and right carotid arteries: common carotid, internal carotid and carotid bulb, in two different angles of insonation. Based on these measurements, the maximum average value of carotid IMT was calculated and expressed in mm ± standard deviation. PWV was measured with the Sphygmocor CPV System (AtCor Medical, Sidney, Australia) [30, 31]. The tonometer was applied at the common carotid artery and the femoral artery. Pulse wave was recorded simultaneously at both locations using electrocardiogram. The latter was chosen as time of reference, enabling the device to determine the transit time of the pulse wave. The distance between the two sites is used to calculate PWV. The device has fully-automated calculations and expresses an index of measurement quality. Per manufacturer recommendations, measures with an index < 74% were not considered. The PWV value given by the apparatus was expressed in meter per second (± standard deviation).

#### Isometric Handgrip Exercise—Magnetic Resonance Imaging (IHE-MRI)

The imaging studies done at baseline (at rest) and during IHE (under stress) for the coronary endothelial function assessment were performed on a clinical 3T MR scanner (MAGNETOM Prisma; Siemens AG, Healthcare Sector, Erlangen, Germany) with an 18-channel chest coil array and a 32-channel spine coil array for signal reception. A 2D radial retrospectively ECG-gated spoiled gradient recalled echo sequence was used to acquire cine cardiac MR images. The relevant imaging parameters included: field of view = 260 × 260 mm^2^, base resolution = 320 sample points per radial line, pixel size = 0.8 × 0.8 mm2, slice thickness = 7.0 mm, echo time = 2.5 ms, repetition time (TR) = 4.7 ms, receiver bandwidth = 580 Hz/pixel, and RF excitation angle = 22 Deg. A product water-selective excitation pulse was used to suppress the signal from epicardial fat and to improve the coronary artery conspicuity. Due to heart rate variation among subjects, the number of readout lines per segment (or views per frame) was adjusted for each subject such that a total of ~250 radial profiles per image were collected within a ~20 s breath-hold duration. The data were acquired with a uniform radial trajectory and 40 cine images were reconstructed at the console. Image quality was rated as poor if blurring due to artifact/patient motion occurred, and as good or very good for optimal image quality.

Maximum grip strength was determined using an MRI-compatible dynamometer (Grip Force Fiber Optic Response Pad, Current Designs Inc., Philadelphia, USA) prior to baseline imaging. Following baseline imaging, IHE-MRI commenced. Handgrip exercise was started one minute prior to data collection. Each subject held the handgrip at 30% of his/her maximum grip strength for approximately 4 minutes during image acquisition. A custom MATLAB software provided real-time feedback to participants enabling them to visually observe grip strength effort while in the magnet—allowing participants to adapt/maintain a constant grip force (30% of maximal effort). Subjects were examined in the supine position and all cine images were acquired during end expiratory breath hold to minimize respiratory motion artifacts.

Bright blood cine MR images were acquired perpendicular to a linear segment of the right/left coronary artery. We used this new technique to measure the changes of diameter of the coronary in response to handgrip exertion, combined to a non-invasive measure of blood pressure and heart rate to determine the rate-pressure product and evaluate the effect of handgrip [20, 32]. As the cross-sectional area of the coronary arteries may change throughout the cardiac cycle [33] measurements at rest and during stress were always made at the same time point in one cardiac cycle, consistent with prior protocols. (18, 20–21, 28). This avoided confounding effects of coronary distensibility (or time phase differences) and minimized effects of motion blurring.

### Statistical analysis

All data were coded before statistical analysis. Assuming normal distribution, paired and non-paired Student-test analysis were performed as appropriate. GraphPadPrim Software was employed to chart data and compare group results. *P* values <0.05 were considered statistically significant.

### Ethics statement

The study protocol was approved by the institutional review board (*Commission cantonale d'éthique de la recherche sur l'être humain [Vaud])* under approval number CERVD 213/15 and was conducted in accordance with the principles of the Declaration of Helsinki.

ClinicalTrials.gov identifier: NCT03506711

Grant information: This study was in part supported by SNF grant 143923.

The authors have declared that no competing interests exist.

## Results

The study included 7 children with T1DM and 16 age- and BMI-matched controls. Characteristics of the study participants are shown in Table 1. Among the patients, the mean duration of T1DM was 10.7 ± 3.1 years, mean total daily insulin 0.9 ± 0.2 U insulin/kg/day, and mean HbA1c 9.3 ± 1.1%. All CIMT and PWV examinations were successfully performed and IHE-MRI examinations were successfully performed in 10/16 (62%) of healthy participants and 6/7 (85%) of patients with T1DM. There were no adverse events. Both CIMT and PWV values were in the expected age-appropriate reference range and no differences were observed between the groups (Table 2). In healthy controls, the mean CIMT and mean PWV were 0.44 ± 0.03 mm and 4.84 ± 0.68 m/s respectively, consistent with normal ranges in prior reports [34–36]. In children with T1DM, mean CIMT (0.46 ± 0.03 mm) and mean aortic PWV (5.33 ± 1.47 m/s) were within a normal range yet slightly but not statistically significantly (both *p* = n.s.) higher than in healthy study participants.

**Table 1:** Characteristics of study participants.

	Healthy Controls (n = 16)	Children with type 1 diabetes (n = 7)	*p*-value
	Mean ± SD	Mean ± SD	
**Age** **(years)**	14.2 ± 2.4	14.8 ± 1.9	0.53
**Height** **(SDS)**	0.49 ± 0.81	-0.09 ± 1.35	0.21
**Weight** **(Kg)**	53.8 ± 13.6	57.7 ± 11.1	0.51
**BMI** **(SDS)**	0.16 ± 0.88	0.6 ± 1.03	0.31
**Pubertal stage (Tanner)**	3.4 ± 1.4	3.9 ± 1.2	0.42
**Heart rate** **at rest** **(/minute)**	72.8 ± 11.1	85 ± 6.9	0.014
**Mean systolic arterial pressure** **at rest** **(mmHg)**	110.6 ± 9	110.7 ± 10.4	0.98
**Mean diastolic arterial pressure** **at rest** **(mmHg)**	64.9 ± 8.8	66.1 ± 12.4	0.793
**Duration of Diabetes** **(years in decimals)**	n/a	10.7 ± 3.1	n/a
**HbA1c** **(%)**	n/a	9.3 ± 1.1	n/a

**Table 2:** Measured values during ultrasound (PWV and CIMT) and IHE-IRM in healthy controls and children with type 1 diabetes mellitus.

	Healthy Controls (n = 16)	Children with type 1 diabetes (n = 7)	*p*-value	Normal values in the litterature
	Mean ± SD	Mean ± SD		Mean / Mean ± SD
**Rate pressure** **product** **Rest** **(bpm x mmHg)**	8248 ± 1549	9478.5 ± 1423.4	0.17	
**Rate pressure** **product** **Handgrip** **(bpm x mmHg)**	11314.9 ± 2356.5	11698.5 ± 886.8	0.757	
**Pulse Wave Velocity** **(PWV)** **(m/s)**	4.84 ± 0.68	5.33 ± 1.47	0.285	Female: 5.113 (3.955 to 6.983) Male: 5.243 (3.640 to 8.021) a)
**Carotid Intima Media Thickness (CIMT)** **(mm)**	0.44 ± 0.03	0.46 ± 0.03	0.173	Healthy controls: 0.44 ± 0.045 Children with T1DM: 0.45 ± 0.054 b)
**Isometric Handgrip Exercise-MRI** **change of area** **(IHE-MRI)** **(%)**	18.84 ± 10.72 (n = 10)	10.5 ± 28.1 (n = 6)	0.013	

a) Values from Reusz GS, Cseprekal O, Temmar M, Kis E, Cherif AB, Thaleb A, et al. Reference values of pulse wave velocity in healthy children and teenagers. Hypertension. 2010;56(2):217–24.

b) Values from Margeirsdottir HD, Stensaeth KH, Larsen JR, Brunborg C, Dahl-Jorgensen K. Early signs of atherosclerosis in diabetic children on intensive insulin treatment: a population-based study. Diabetes Care. 2010;33(9):2043–8.

### IHE-MRI

**Fig 1:**
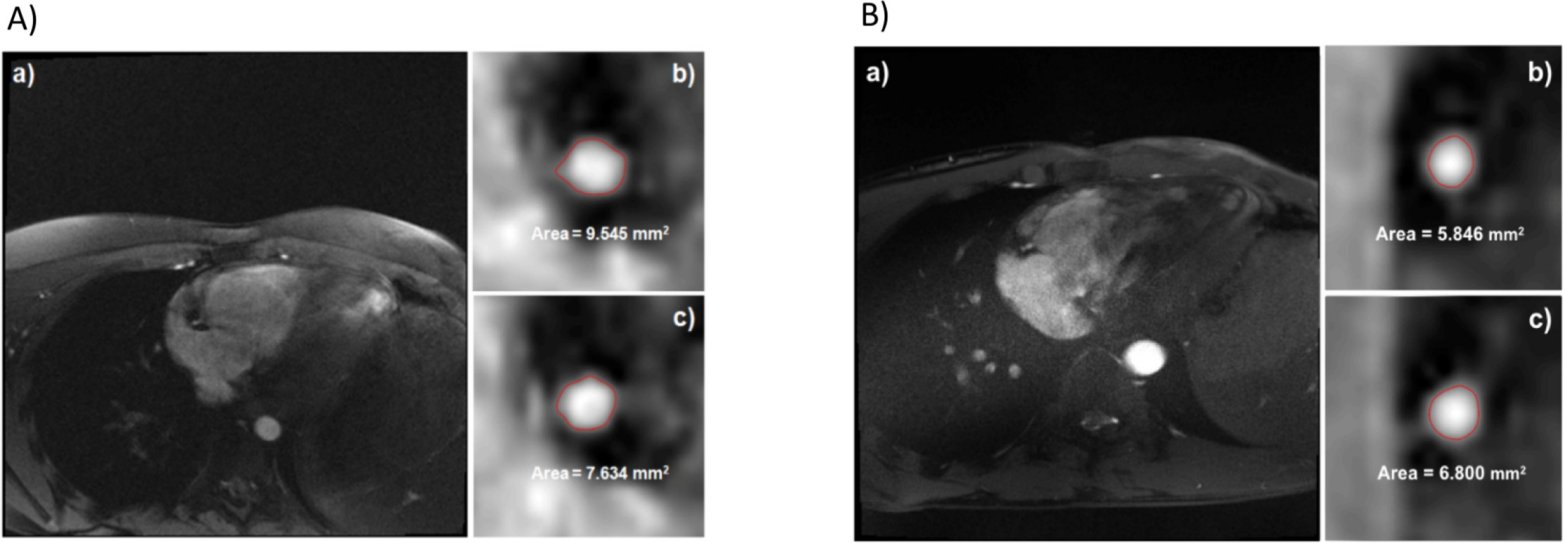
Representative MR images. Representative MR anatomical and flow velocity images during measurement of coronary vasomotor response of the right coronary artery (RCA) to handgrip exercise. Representative images obtained from a child with A) T1DM (left panel) and B) a healthy control (right panel). (a) double oblique scout scan obtained in parallel to the RCA. (b) cross-sectional images of the RCA acquired at rest (baseline). (c) cross-sectional images of the RCA during isometric handgrip stress. The vessel lumen area is represented by the red line.

**Fig 2:**
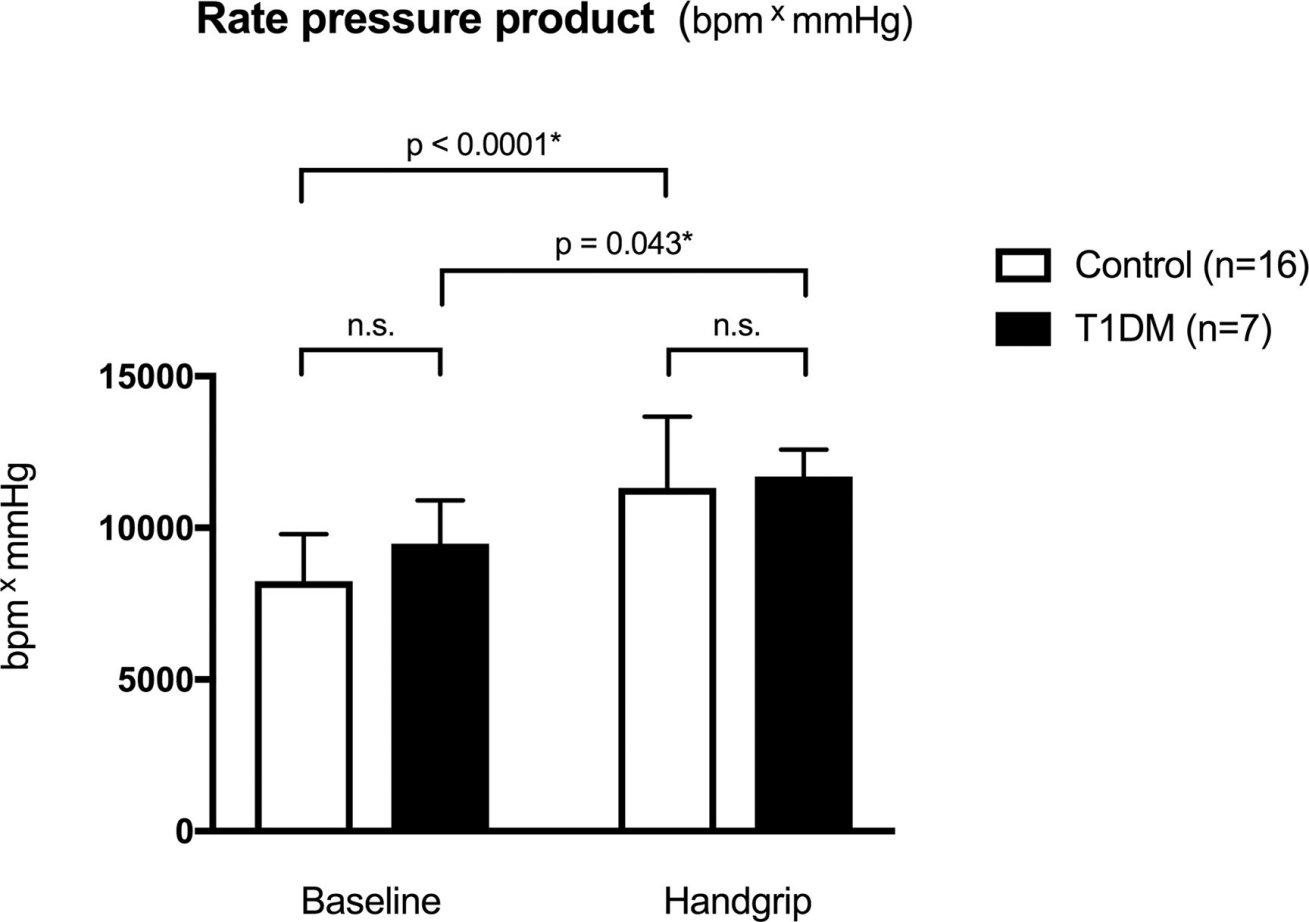
**Hemodynamic effects of exercise during IHE-MRI measured by rate pressure product change in healthy (white boxes) and children with type 1 diabetes mellitus (black boxes).** Both groups show significant increase in the rate pressure product during handgrip confirming the usability of the IHE-method to induce stress. Children with T1D had a higher but not significant baseline rate pressure product.

The IHE-MRI was feasible for children and young adults as evidenced by the fact that good to very good vascular image quality (i.e. >75% per manufacturer recommendation) was obtained in 6/7 (85%) of patients with T1DM and 10/16 (62%) of healthy participants (Fig 1). Technical reasons for low quality IHE-MRI were varied: poor ECG triggering (n = 2), increased heart rate (n = 2), inaccurate planning of slice position, increased magnetohydrodynamic effects and anatomical and physiological variations (e.g. coronary artery size), absence of quiescent period during diastole, as well as noncompliance of subjects (n = 1 each). IHE-MRI technique identified significant changes of blood pressure and rate pressure product (Fig 2). In the healthy group, we observed a mean + 37% increase in the rate pressure product (RPP) during handgrip, and a + 23.4% increase in the T1DM group. These data confirm the usability of the IHE-method to induce stress under MRI in children and adolescents.

### Comparison of IHE-MRI: T1DM versus healthy controls

**Fig 3:**
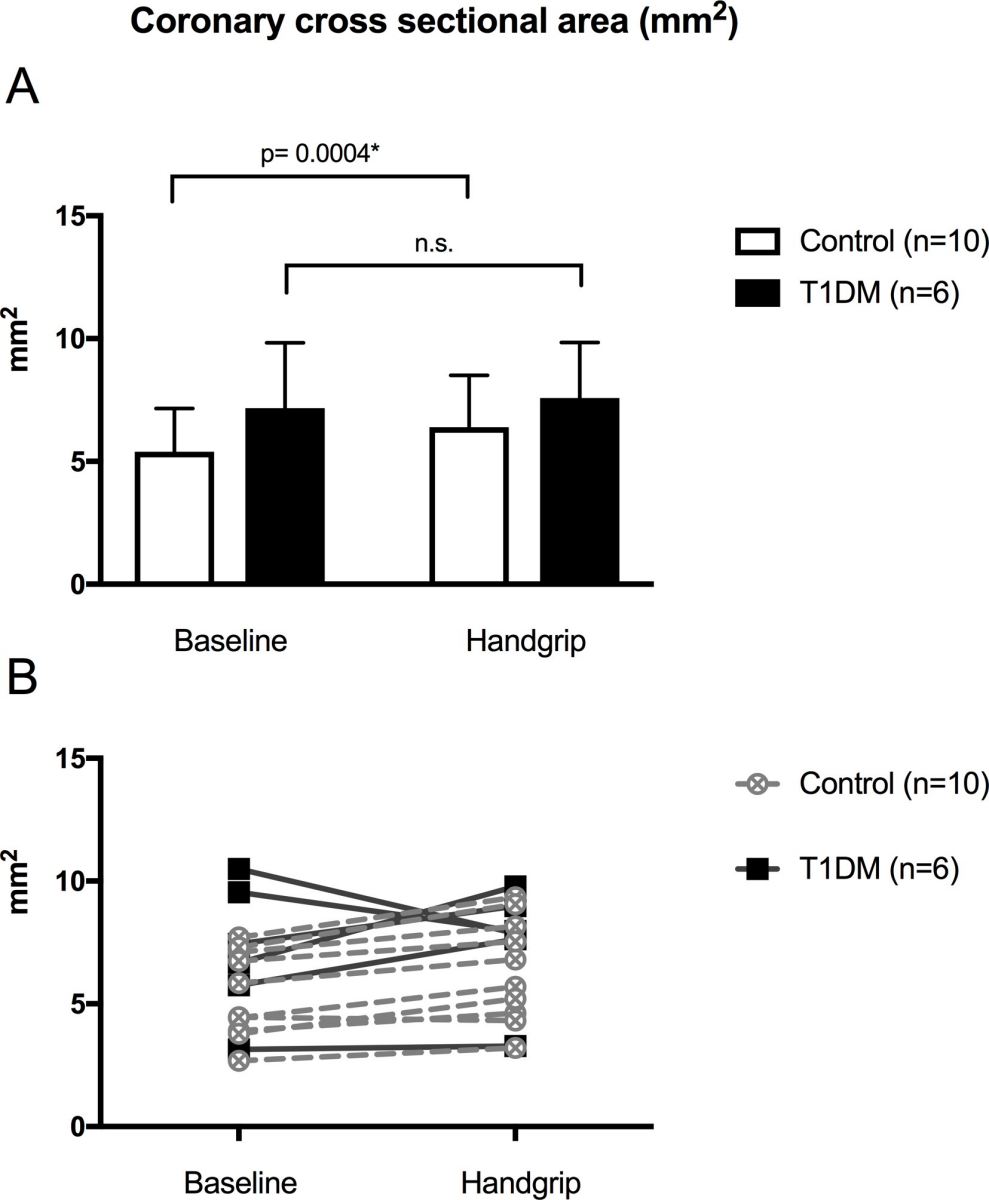
Comparison of changes in coronary cross-sectional area during IHE. The white boxes represent the mean volumetric change in mm^2^ of coronary artery area in healthy children. A: The black boxes represent the mean volumetric change in mm^2^ of coronary artery area in patients with Type 1 diabetes mellitus (T1DM). B: individual changes in coronary cross-sectional area.

Only good or very good quality images (n = 16) were used in this specific analysis. A significant increase in coronary cross-sectional area was observed in healthy controls (mean: 5.4 mm^2^ at rest to 6.39 mm^2^ under stress (percent change 18.8 ± 10.7%, *p* = 0.0004), consistent with the expected vasodilatory effect of the IHE. In contrast, no significant difference in average cross-sectional coronary area change between rest and during IHE was observed in children with T1DM (mean: 7.17 mm^2^ at rest to 7.59 mm^2^ under stress, 10.5% ± 28.1%, n.s.) (Fig 3). These observations are consistent with a blunted endothelium dependent response.

## Discussion

Results from this pilot study demonstrate the initial feasibility of using a totally non-invasive IHE-MRI technique in children and adolescents with and without T1DM. Compared to baseline measurements, the differences in blood pressure and rate pressure product during handgrip suggest the utility of this safe and non-invasive technique as an endothelium-dependent stressor. As the release of endothelium derived nitric oxide is thought to be necessary to regulate vascular response to blood flow demands during exercise [20, 37], the vasomotor response observed in the coronary arteries of healthy subjects suggests that this technique can be used as a marker of endothelial vascular function in children and adolescents—corroborating findings in healthy adults [38, 39].

Although the sample size is limited, our data suggest a decrease/absence of change in cross-sectional area in response to handgrip in patients with T1DM (Fig 3). This observation could be consistent with a blunted endothelium-dependent response in contractile tone that could result from decreased nitric oxide production [40, 41]. This blunted response has been observed in aged subjects and patients with essential hypertension [40], but has not previously been described in children with T1DM.

### Comparison to results in adults

Abnormalities in endothelium-dependent coronary vasomotor function have clearly been linked to development and progression of coronary artery disease (CAD) in adults, provide a marker for subclinical disease, and represent an independent predictor of adverse cardiac events (18,23–25).

Our results compare well to recently published findings in an adult cohort, where increase of hemodynamic responses in healthy adults of a mean +35.4% was reported (+37% in our group) [21]. In the same study, cross sectional coronary area changes were reported at +12% in n = 26 healthy adult, comparing well to the +18.84% found in the healthy subjects of our cohort.

High-quality IHE-MRI images suggest that we could successfully differentiate endothelium-dependent cross-sectional coronary area changes in response to stress–a finding not previously reported in a pediatric population.

### Differences detected by IHE-MRI technique

CIMT values and PWV values in our study were in the expected normal range and no differences were found between groups during handgrip exertion. In contrast, IHE-MRI technique suggested differences between groups in terms of area changes (baseline vs. stressed) pointing to an early impact of diabetes on endothelial function. This finding corroborates observations made in a cohort of adult patients with coronary artery disease, where no vasodilatation was measured in response to IHE [21].

### Limitation of the present study

The relatively high levels of variance reported herein draws attention to several technical issues. First, the RCA is often tortuous and a perfectly straight segment cannot always be found. However, and while this may have an impact on area measurements, this likely affects both rest and stress measurements the same way and may not affect percent area changes that much. Second, the accuracy and precision of the ECG triggered signals was worse during exercise than at rest. These observations raise the question if this results from the increase in heart rate under stress. Images were indeed more blurred under stressed conditions compared to those at rest (baseline), however, since cine images were acquired with a high temporal resolution, the effects of motion blurring could be minimized. Still, rapid cardiac motion consistent with stress and higher heart rates in children may have to be considered. Hays and colleagues demonstrated that vasodilation during handgrip exercise only occurs in healthy adult subjects in control conditions with standard ECG-STD acquisitions and not during L-NMMA infusion (to inhibit nitric oxide) [20].

Additional studies will be needed to clarify questions of imaging definition by including participants with different coronary cross-sectional area. Second, the act of using the handgrip may have influenced image quality. A number of participants moved their upper body during exertion using the handgrip. Thus, brief orientation/training on handgrip use without moving the upper body or the arm before entering the MRI seems both relevant and necessary. Alternatively, investigating the study subjects in the prone position as suggested by Hays et al. may help mitigate that problem at the expense of patient comfort. Training young patients to hold their breath during imaging may be another strategy to improve the success rate. The IHE protocol employed a unilateral handgrip. An alternative may be to develop a bilateral, symmetrical handle that is fixed to the MRI bed–not for measuring grip strength per se, but rather to limit upper limb movements. This would entail updating the IHE-MRI protocol and adapt it for children.

### Further studies

In this preliminary study in children and adolescents, we observed relatively large standard deviations for mean difference in average cross-sectional coronary area (mean 1.0 mm^2^ (SD 0.57) in volunteers, and mean 0.41 mm^2^ (SD 2.2) in T1DM). This observation is likely due to the limited sample size of this pilot study. A *post hoc* power calculation indicates that 37 healthy volunteers and 141 patients with T1DM would be required to identify statistically significant differences with a power of 80% and alpha-level of 0.05 (two-sided). We presume that by including young adult subjects up to 25 years of age with larger coronary arteries and lower resting heartrate will help ameliorate some of the image quality concerns. Further, the sample size would be sufficient to identify statistically significant differences in cIMT and PWV–based on data reported herein (cIMT: 0.440±0.027mm, PWV: 4.84 ± 0.68 m/s).

The preliminary pilot imaging results reported herein suggest reduced endothelial function in children with T1DM compared to healthy controls. Differences were identified using the novel IHE-MRI protocol but not using standard CIMT and PWV exams. There are several limitations to this study including small sample size and the fact that not all images obtained were high quality. One possible bias is that investigators were not blinded to the participants’ condition and that normal dilatation with handgrip was expected in healthy controls while milder effects were anticipated among children with T1DM. As this was a pilot feasibility study, it is not powered to detect significant differences, and further studies are needed to confirm this finding and to relate it to disease duration and severity. The absence of ionizing radiation will permit serial studies to measure progression/regression and help to monitor therapy, which is not ethically justifiable with any invasive method or CT. As it is quantitative and precise, such serial studies are critically supported.

## Conclusions

Data from the present study support the IHE-MRI as a safe, non-invasive and sensitive tool for detecting coronary vascular complications in children and adolescents with T1DM. The IHE-MRI technique described herein is feasible and is capable of producing high-quality images. The observed differences in vascular response to IHE in children and adolescents with T1DM compared to healthy controls were not identified by the clinically available CIMT and PWV techniques. This novel use of IHE-MRI method in children may enable visualization of vascular effects and changes in endothelial function—an early manifestation of coronary atherosclerosis. Such an advance could help improve care for high risk patient populations.

## Supporting information

S1 File Summary of coronary cross-sectional area measurements. (XLSX)

S1 Table (XLSX)

S2 Table (XLSX)
